# Decision-tree-based ion-specific dosing algorithm for enhancing closed hydroponic efficiency and reducing carbon emissions

**DOI:** 10.3389/fpls.2023.1301490

**Published:** 2023-12-18

**Authors:** Woo-Jae Cho, Min-Seok Gang, Dong-Wook Kim, JooShin Kim, Dae-Hyun Jung, Hak-Jin Kim

**Affiliations:** ^1^ Department of Biosystems Engineering, College of Agriculture & Life Sciences, Gyeongsang National University, Jinju, Republic of Korea; ^2^ Institute of Smart Farm, Gyeongsang National University, Jinju, Republic of Korea; ^3^ Department of Biosystems Engineering, College of Agriculture and Life Sciences, Seoul National University, Seoul, Republic of Korea; ^4^ Integrated Major in Global Smart Farm, College of Agriculture and Life Sciences, Seoul National University, Seoul, Republic of Korea; ^5^ Department of Smart Farm Science, Kyung Hee University, Yongin, Republic of Korea

**Keywords:** ion-selective electrodes, ion-specific replenishment, decision tree, closed hydroponics, dosing algorithm, carbon dioxide emissions

## Abstract

The maintenance of ion balance in closed hydroponic solutions is essential to improve the crop quality and recycling efficiency of nutrient solutions. However, the absence of robust ion sensors for key ions such as P and Mg and the coupling of ions in fertilizer salts render it difficult to effectively manage ion-specific nutrient solutions. Although ion-specific dosing algorithms have been established, their effectiveness has been inadequately explored. In this study, a decision-tree-based dosing algorithm was developed to calculate the optimal volumes of individual nutrient stock solutions to be supplied for five major nutrient ions, i.e., NO_3_, K, Ca, P, and Mg, based on the concentrations of NO_3_, K, and Ca and remaining volume of the recycled nutrient solution. In the performance assessment based on five nutrient solution samples encompassing the typical concentration ranges for leafy vegetable cultivation, the ion-selective electrode array demonstrated feasible accuracies, with root mean square errors of 29.5, 10.1, and 6.1 mg·L^-1^ for NO_3_, K, and Ca, respectively. In a five-step replenishment test involving varying target concentrations and nutrient solution volumes, the system formulated nutrient solutions according to the specified targets, exhibiting average relative errors of 10.6 ± 8.0%, 7.9 ± 2.1%, 8.0 ± 11.0%, and 4.2 ± 3.7% for the Ca, K, and NO_3_ concentrations and volume of the nutrient solution, respectively. Furthermore, the decision tree method helped reduce the total fertilizer injections and carbon emissions by 12.8% and 20.6% in the stepwise test, respectively. The findings demonstrate that the decision-tree-based dosing algorithm not only enables more efficient reuse of nutrient solution compared to the existing simplex method but also confirms the potential for reducing carbon emissions, indicating the possibility of sustainable agricultural development.

## 1 Introduction

In the application of closed hydroponic solutions, the maintenance of ion balance in nutrient solutions is fundamental not only to ensure the productivity and quality of crops but to prolong the recycling period of the nutrient solution. This helps reduce the water and nutrient discharge, thereby providing economic and environmental benefits (Bamsey et al., 2012; Sambo et al., 2019). However, most soilless cultivation systems replenish the nutrient solution based on the pH and electrical conductivity of the solutions, without considering the varying concentrations of individual ions (Geoffrey et al., 1997; Domingues et al., 2012; Katsoulas et al., 2015; Kozai et al., 2018; Son et al., 2020). Furthermore, nutrient uptake rates vary with the growth and development of plants, adding complexity to the management of nutrient composition in recycled nutrient solutions (Sambo et al., 2019; Ahn et al., 2021). Consequently, the ion concentrations in the nutrient solution may deviate from the optimal composition, potentially compromising the crop yield and quality. In addition, excessive nutrient solution application may lead to inefficient carbon emissions.

Despite variations in the uptake speed of each nutrient ion, the average nutrient uptake can be estimated by monitoring changes in water and nutrients in the recirculated nutrient solution through a mass balance equation (Neocleous and Savvas, 2016). Achieving nutrient balance in the recycled nutrient solution involves supplementing deficient ions to reach the target concentration for each nutrient (Neocleous and Savvas, 2022). Consequently, the timely measurement and replenishment of the nutrient solution are crucial tasks for optimizing closed hydroponic systems.

Recently, ion-specific nutrient management based on ion-selective sensors has been investigated, and its potential in hydroponic applications has been demonstrated (Rius-Ruiz et al., 2014; Vardar et al., 2015; Jung et al., 2019; Xu et al., 2020). Furthermore, several researchers have developed automated nutrient management systems using ion-selective electrodes (ISEs) that can measure the concentrations of individual ions in hydroponic solutions and then adjust the nutrient dosages according to deficient ions (Gieling et al., 2005; Jung et al., 2015; Cho et al., 2017; Jung et al., 2019; Xu et al., 2020). However, it remains challenging to realize ion-specific management for all nutrient ions using ion sensors due to the scarcity of robust ISEs for crucial ions such as P and Mg. Consequently, the existing research on automated ion-specific nutrient management has been limited to only certain major ions. Furthermore, these studies have seldom considered the possibility that nutrient salts may include other ions that cannot be measured by sensors or that are not consumed by plants.

For example, Xu et al. (2019) recommended the introduction of a concentrated KCl solution when the K concentration measured by a K ISE dropped below a certain threshold. Although this approach is simple and cost-effective, it does not consider the coupled injection of Cl ions, which may be harmful for crop growth (Shiyab et al., 2013). Later, Xu et al. (2020) improved their system by using additional ISEs of K, NO_3_, and H_2_PO_4_ for managing the nutrient solution. However, Na or SO_4_ ions were still introduced during the replenishment based on NaNO_3_, NaH_2_PO_4_, and K_2_SO_4_. In this context, the ion coupling must be comprehensively examined for alleviating the adverse effects on plants in closed hydroponics.

A simplex algorithm, which simultaneously calculates the injection volumes of stock solutions subject to certain constraints, can facilitate the accurate injection of nutrient ions (De Rijck and Schrevens, 1994; Gieling et al., 2005; Jung et al., 2015). Specifically, this algorithm computes the amounts of fertilizer solutions to be added by performing matrix calculation that consider the contribution ratios and concentrations of the nutrient ions contained in the fertilizer solutions. It can theoretically obtain the complete solution for given constraints. However, in practical hydroponic applications, the solution may include negative values that cannot be achieved by nutrient dosing systems.

As an alternative, Cho et al. (2017) and Jung et al. (2019) proposed a sequential calculation method based on predetermined priorities of the ions to minimize the inevitable injection of nutrient ions. This approach used six fertilizers to mitigate the problem of decoupled replenishment among nutrients, with the P and Mg ions managed by applying linear concentration ratios related to NO_3_ and Ca ions, respectively. However, this algorithm did not account for NH_4_; micronutrients, such as Fe, Zn, and Cu; and the water volume. In addition, the control logic of injecting fertilizer solutions after water replenishment resulted in inaccurate and inefficient supplementation. Thus, the development of an improved fertilizer dosing algorithm that can robustly maintain individual ion concentrations at the required levels while minimizing accumulations or deficiencies of unmeasurable ions is imperative.

Considering these aspects, in this study, NO_3_, K, and Ca ISEs were used for monitoring the ion concentrations in recycled nutrient solutions, and a decision-tree-based dosing algorithm was established to determine the proper amounts of fertilizers while minimizing the coupled injection of nutrient ions. Variable ion-specific replenishment was achieved by controlling the operation time of individual fertilizer pumps. These procedures were automated through an ion-specific nutrient management system capable of both ion-specific monitoring and replenishment. The ion-monitoring performance was evaluated by comparing the determined concentrations with those obtained using standard analytical methods. The decision-tree-based dosing algorithm was validated through a five-step replenishment simulation test. The fertilizer injections and resulting concentrations were compared with those obtained using the simplex method using the same ion monitoring and fertilizers. Additionally, carbon dioxide emissions from both methods were compared to assess the environmental impact.

## 2 Materials and methods

### 2.1 Decision-tree-based dosing algorithm

The relative proportion of these ions must be considered because fertilizer salts can dissolve into more than two ions. Although the use of various fertilizers can enable flexible control of individual ion concentrations, practical challenges exist. For example, the supply of Ca ions cannot be decoupled from NO_3_ ions owing to the absence of alternative fertilizer salts (Resh, 2016). In addition, the use of multiple fertilizers would require larger tank spaces and increase the complexity of the calculation and system operation. Therefore, in this study, seven fertilizers, including Ca(NO_3_)_2_·4H_2_O, KH_2_PO_4_, NH_4_H_2_PO_4_, KNO_3_, NH_4_NO_3_, MgSO_4_·7H_2_O, and K_2_SO_4_, were selected as stock solutions to ensure that at least two salts are available for each ion, except for Ca and Mg. Subsequently, the priority of ions was determined based on the universal nutrient-solution calculation method, i.e., Ca > P = K > NO_3_ > NH_4_ (Sonneveld et al., 1999). In practice, various physical-chemical phenomena can affect nutrient availability for plants, especially precipitation and complexation, which are closely related to pH and nutrient solution temperature (De Rijck and Schrevens, 1994; Sambo et al., 2019). However, this study assumes that the pH and the temperature of hydroponic solutions are maintained consistently in controlled environment, concentrating solely on mass balance considerations.

To calculate the appropriate mass of the fertilizer salts based on the given ion concentrations and priority, a decision tree was used. The decision tree method is a machine‐learning method for constructing a series of dichotomous classifications (Namazkhan et al., 2020). The algorithm creates tree-shaped diagrams with a number of branches with decision and leaf nodes. Each decision node has a predictor variable to obtain a more accurate response for the given variable, and the leaf node represents the final optimized result within the decision tree model. The decision-tree-based dosing algorithm consists of three parts. The first part involves the calculation of the amounts of major ions considering the current nutrient solution volume, target nutrient solution volume, and ion compositions in water (Equations 1–6). SO_4_ is not considered because it is not harmful to crops (Sonneveld et al., 1999).


(1) $$N_{Ca}=T_{Ca}\times V_{target}-D_{Ca}\times V_{current}-W_{Ca}\times \left(V_{target}-V_{current}\right)$$

(2) $$N_{K}=T_{K}\times V_{target}-D_{K}\times V_{current}-W_{K}\times \left(V_{target}-V_{current}\right)$$

(3) $$N_{NO3}=T_{NO3}\times V_{target}-D_{NO3}\times V_{current}-W_{NO3}\times \left(V_{target}-V_{current}\right)$$

(4) $$N_{NH4}=R_{N-N}\times N_{NO3}-W_{NH4}\times \left(V_{target}-V_{current}\right)$$

(5) $$N_{Mg}=T_{Mg}\times V_{target}-C_{Mg}\times V_{current}-W_{Mg}\times \left(V_{target}-V_{current}\right)\;,\text{ or }R_{Ca-Mg}\times N_{Ca}-W_{Mg}\times \left(V_{target}-V_{current}\right)$$

(6) $$N_{P}=T_{P}\times V_{target}-C_{P}\times V_{current}-W_{P}\times \left(V_{target}-V_{current}\right),\text{ or }R_{N-P}\times N_{NO3}-W_{P}\times \left(V_{target}-V_{current}\right)$$

where

*N*_x_ = amounts of ions (*x* = Ca, K, NO_3_, NH_4_, Mg, or P) to be replenished (mg).

*T*_x_ = target concentrations of ions (*x* = Ca, K, NO_3_, NH_4_, Mg, or P).

*D*_y_ = concentrations of ions (*y* = Ca, K, or NO_3_) determined by ISEs (mg·L^-1^).

*W*_x_ = concentrations of ions (*x* = Ca, K, NO_3_, NH_4_, Mg, or P) in water determined by standard analyzers (mg·L^-1^).

*V*_target_ = target volume of the nutrient solution in the mixing tank (L).

*V*_current_ = current volume of the nutrient solution in the mixing tank (L).

*C*_z_ = concentrations of ions (z = Mg or P) determined by standard instruments.

*R*_N-N_, *R*_Ca-Mg_, *R*_N-P_ = absorption ratios of NO_3_ to NH_4_, Ca to Mg, and NO_3_ to P, respectively.

**Figure 1:**
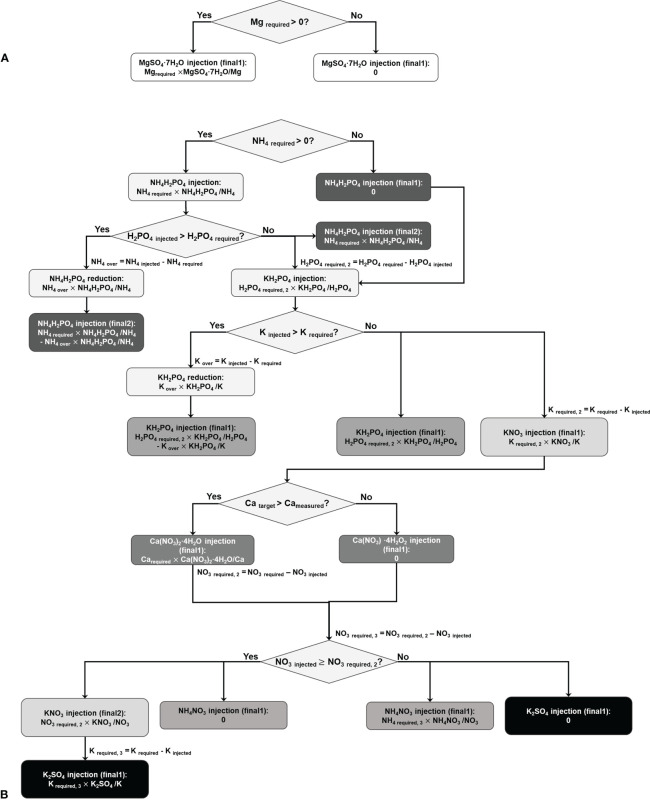
Decision tree model for calculating the amounts of the fertilizer salts to be replenished, for Mg(SO_4_)_2_·7H_2_O **(A)** and other salts **(B)**. The X_injected_ (X: NH_4_, H_2_PO_4_, K, or NO_3_) represents the injected amount of the ion by the previously injected salt. The node including ‘final’ indicates the leaf node, and the higher number behind the ‘final’ means the result would be a more appropriate amount of the salt.

The second part is the decision-tree-based calculation of the required amounts of fertilizer salts while minimizing over-injection. **Figure 1** illustrates the calculation process, in which the algorithm contains two trees.

The first tree calculates the proper mass of Mg(SO_4_)_2_·7H_2_O. Given that there exists only one salt for Mg replenishment and the injection of Mg(SO_4_)_2_·7H_2_O does not affect the other nodes, this tree operates independently (**Figure 1A**).

The second tree calculates the amounts of the other salts, i.e., Ca(NO_3_)_2_·4H_2_O, KH_2_PO_4_, NH_4_H_2_PO_4_, KNO_3_, NH_4_NO_3_, and K_2_SO_4_. These salts are interconnected with one another, and thus, the tree categorizes the salts according to predetermined priorities. Next, the amounts of salts are sequentially calculated. For example, if NH_4_ is required to be replenished, the amount of NH_4_H_2_PO_4_ is calculated based on the required mass of NH_4_. The subsequent node assesses the effect of the calculated amount of NH_4_H_2_PO_4_ in H_2_PO_4_. If H_2_PO_4_ is not overdosed, NH_4_H_2_PO_4_ is injected as calculated. If not, the amount of NH_4_H_2_PO_4_ to be supplied is re-calculated based on the required amount of H_2_PO_4_ because the priority of P is higher than that of NH_4_. In this case, the second final amount of the NH_4_H_2_PO_4_ is administered rather than its first final amount. Similarly, the decision-tree-based approach is used to calculate the amounts of other salts (**Figure 1B**).

After determining the amounts of salts to be supplied, the runtime of the pump corresponding to each fertilizer salt is obtained using Eq. 7.


(7) $$P_{x}=\frac{M_{x}}{C_{x}\times D_{x}}$$

where

*x* = Ca(NO_3_)_2_·4H_2_O, KH_2_PO_4_, NH_4_H_2_PO_4_, KNO_3_, NH_4_NO_3_, MgSO_4_·7H_2_O, or K_2_SO_4_.

*P*_x_ = runtime of metering pump for stock solution of fertilizer salt *x* (s).

*M*_x_ = mass of stock solution of fertilizer salt *x* (mg).

*C*_x_ = concentration of stock solution of fertilizer salt *x* (mg·L^-1^).

*D*_x_ = discharge volume of metering pump for seven stock solutions of fertilizer salts (L·s^-1^).

The third part of the dosing algorithm focuses on micronutrients and water replenishment. At present, only a few ionophores are commercially available for micronutrient ions. Therefore, micronutrients are replenished by injecting micronutrients proportional to the difference between the target and current volumes of the nutrient solution (Eq. 8).


(8) $$P_{m}=\frac{C_{m}\times \left(V_{target}-V_{current}\right)}{D_{m}}$$

where

*P*_m_ = runtime of metering pump for concentrated solution of micronutrients (s).

*C*_m_ = multiple of concentrated solution of micronutrients to the final working concentration (dimensionless).

*D*_m_ = discharge volume of metering pump for concentrated solution of micronutrients (L·s^-1^).

Then, the volume of water to add can be obtained by subtracting the total volumes of the stock solutions and concentrated micronutrient solution from the difference between the target and current volumes of the nutrient solution (Eq. 9).


(9) $$P_{w}=\frac{V_{target}-V_{current}-\sum V_{\mathrm{stock}\;\mathrm{solution}\;\mathrm{for}\;x}-V_{m}}{D_{w}}$$

where

*x* = Ca(NO_3_)_2_·4H_2_O, KH_2_PO_4_, NH_4_H_2_PO_4_, KNO_3_, NH_4_NO_3_, MgSO_4_·7H_2_O, or K_2_SO_4_.

*P*_w_ = runtime of metering pump for water (s).

*V*_stock solution for x_ = volume of stock solution of fertilizer salt *x* to be added (L).

*V*_m_ = volume of concentrated solution of micronutrients to be added.

*D*_w_ = discharge volume of metering pump for water (L·s^-1^).

### 2.2 Development of an ion-specific nutrient management system

**Figure 2:**
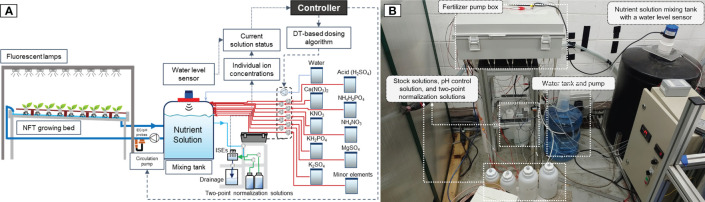
Schematic **(A)** and picture **(B)** of the ion-specific nutrient management system.

The ion-specific nutrient management system must be able to automatically measure the ion concentrations of the nutrient solution, replenish the nutrient solution considering the ion balance, and supply the optimal nutrient solution to the growing bed. **Figure 2** shows a schematic and an image of the ion-specific nutrient management system.

For the solutions used by the system, a nutrient mixing tank and twelve reservoirs were introduced. These reservoirs corresponded to the seven fertilizer stock solutions, one micronutrient stock solution, one pH control solution, water, and two-point normalization solutions (**Figure 2**). For two-point normalization, two mixed solutions containing NO_3_, K, and Ca ions at two different concentrations, i.e., 100 and 1,000 mg·L^−1^, 30 and 300 mg·L^−1^, and 26 and 260 mg·L^−1^, respectively, were prepared based on the composition of the modified Hoagland’s hydroponic nutrient solution to minimize the background effects from the real hydroponic solutions (Hoagland and Arnon, 1950; Cho et al., 2019). The ion concentrations of the prepared stock solutions, pH control solution, and two-point normalization solutions are presented in **Table S1**.

To monitor the volume of the nutrient solution tank, a reflective ultrasonic water-level transmitter (EchoPod UG01, Flowline, Inc., CA, USA) was installed on the mixing tank (**Figure 2**).

For the two-point normalization process, as well as for sampling, drainage, and administration of stock solutions, peristaltic pumps were employed due to their advantages, such as ability of ensuring sanitized transport of the fluid, self-priming operation, absence of backflow, and high repeatability (Klespitz and Kovács, 2014). In general, the flow rate of each peristaltic pump determines the minimum injection volume, which is important as it directly affects the accuracy of replenishment. Therefore, the flow rates of the pumps for stock solutions and water were determined to maintain relative errors from the minimum injection volume of less than 0.1%. This value was set considering that the stock solutions were prepared with concentrations of 20,000 mg·L^-1^, and the multiple of the concentrated minor element solution was 200. To ensure chemical resistance, novoprene tubing was used for the injection pumps (SR10/50, ASF THOMAS, Puchheim, Germany) of fertilizer salts (i.e., Ca(NO_3_)_2_·4H_2_O, KH_2_PO_4_, NH_4_H_2_PO_4_, KNO_3_, NH_4_NO_3_, MgSO_4_·7H_2_O, and K_2_SO_4_), micronutrients, and acid, considering their high concentrations. Similarly, PharMed BPT tubing was applied to the pumps for the two-point normalization solutions. The drainage, sampling, and water pumps used silicone tubing owing to the lower concentrations of ions.

To quantify the NO3 and K ions, ISEs using two different polyvinyl chloride (PVC)-based ion-selective membranes were fabricated according to chemical compositions and procedures reported in previous studies (Kim et al., 2013; Jung et al., 2015; Cho et al., 2017). A commercially available Ca ISE (Orion 9320BN, Thermo Fisher, MA, USA) was used to measure the Ca ion concentrations. Finally, an array of ISEs composed of three ISEs for NO_3_, three ISEs for K, two ISEs for Ca, and one reference electrode was installed in a sample chamber for measuring the ion concentrations in the nutrient solutions. A double-junction electrode (Orion 900200, Thermo Fisher, MA, USA) was used as the reference electrode. To minimize the presence of residual solutions following drainage, which could induce measurement errors, the bottom of the sensor array chamber was designed to have a slope of 15° to facilitate drainage.

An isolation circuit board (NI SCC-AI13, National Instruments, TX, USA) was used to buffer the impedance of each electrode, and the buffered signals were collected using a data acquisition board (NI PCI-6221, National Instruments, TX, USA).

The system specifications are listed in **Table S2**.

### 2.3 Sensor performance test

The performance of the ion sensors in the system was validated through a measurement test involving five nutrient solution samples that encompassed the typical concentration ranges for leafy vegetable cultivation. Specifically, the test solutions were derived from real hydroponic solutions used during lettuce cultivation, prepared based on the modified Hoagland’s hydroponic nutrient solution recipe (Hoagland and Arnon, 1950).

During the test, the system conducted a series of measurements following the sampling and measurement processes. The two-point normalization solutions were prepared to have NO_3_, K, and Ca ions at two different concentrations (100 and 1,000 mg·L^-1^, 30 and 300 mg·L^-1^, 24 and 240 mg·L^-1^, respectively) with the same background components as the nutrient solution.

After each measurement, the nutrient solution was sampled and analyzed using a commercial soil and water quality analysis center (NICEM, Seoul, South Korea) to determine actual concentrations using standard analyzers, i.e., ion chromatography for NO_3_ and inductively coupled plasma (ICP) spectrophotometry for K and Ca measurements. Subsequently, the performance of the ion sensors was evaluated by comparing the concentrations determined by the sensors and standard methods.

### 2.4 Dosing algorithm validation

The system performance was validated through a five-step management test. Specifically, the test began with a mixture of the modified Hoagland’s hydroponic nutrient solution (Hoagland and Arnon, 1950). Next, the system conducted a series of nutrient adjustments according to the given target concentrations of NO_3_, K, and Ca, with increasing levels of the target nutrient solution volume. The target concentrations were randomly established at three levels: 80%, 100%, and 120% of the standard concentrations. The target values for the stepwise management test are summarized in **Table 1**. After each replenishment, the nutrient solution was sampled and analyzed by the commercial soil and water quality analysis center (National Instrumentation Center for Environmental Management, Seoul, South Korea) to determine actual concentrations using standard analyzers, i.e., ion chromatography for NO_3_ and ICP spectrophotometry for K and Ca measurements. Subsequently, the performance of the replenishment sequence was evaluated by comparing the target and actual concentrations determined by standard methods.

**Table 1:** Target values of hydroponic solutions to be supplied in the stepwise test.

Step	Target ion concentration (mg·L^-1^)			Target water volume (L)
	Ca	NO_3_	K	
Initial	80	434	117	10
1^st^	80	347.2	93.6	15
2^nd^	96	347.2	117	20
3^rd^	64	434	140.4	25
4^th^	80	434	93.6	30
5^th^	96	520.8	140.4	40

The automated ion-specific nutrient management was executed with lower limits of 20% and 10% for the ion concentrations and nutrient solution volume, respectively, to facilitate closed-loop control. The two-point normalization solutions were prepared to have NO_3_, K, and Ca ions at two different concentrations (100 and 1,000 mg·L^-1^, 30 and 300 mg·L^-1^, 24 and 240 mg·L^-1^, respectively) with the same background components as the nutrient solution.

To evaluate the performance of the proposed dosing algorithm, simulated calculations for the ion concentrations during the stepwise test were conducted based on the conventional simplex matrix method (Gieling et al., 2005; Jung et al., 2015).

### 2.5 Evaluation of carbon dioxide emissions

Carbon dioxide emissions from fertilizer salts vary according to the region and fertilizer type because fertilizer companies typically use different fertilizer production technologies and stocks (Brentrup et al., 2018). Therefore, it is challenging to accurately determine carbon dioxide emissions from fertilizers unless all the production details are available. Thus, in this study, the carbon dioxide emissions were assumed for comparing the environmental impact of two types of fertilizer dosing algorithms. From the injected amounts of fertilizer salts, the potential of CO_2_ equivalent (g CO_2_eq/g) was calculated according to the results of Wang et al. (2017) for N (1.526 g CO_2_eq/g), K (0.6545 g CO_2_eq/g), and P (1.631 g CO_2_eq/g).

## 3 Results

### 3.1 Sensor performance test

**Figure 3:**
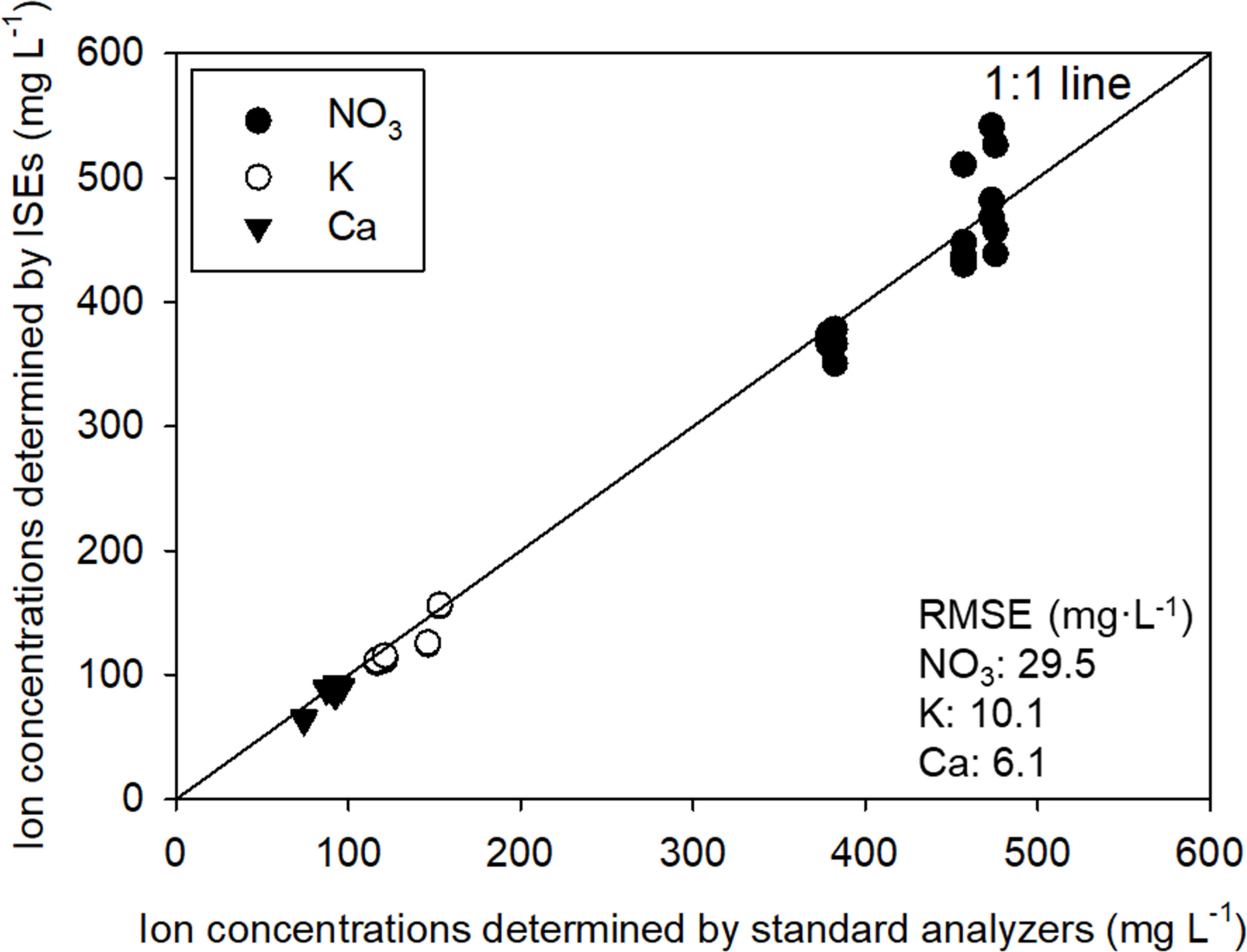
Comparison of ion concentrations in the resulting solutions of the stepwise test predicted by standard analysis and ISEs.

The feasibility of the ion concentration measurements obtained by the system using the ISEs were assessed through a comparison with those determined using standard analyzers (**Figure 3**). The measured ion concentrations were examined at five-step validation solution levels for Ca, K, and NO_3_. Formed based on the concentration range of the nutrient solution composition commonly used, NO_3_, K, and Ca ISE all exhibited measured values closely resembling the actual values. As a result, the measured and actual values were close to unity. In terms of the root mean square errors (RMSEs), the accuracies of the ISE array measurements were 29.5, 10.1, and 6.1 mg·L^-1^ for NO_3_, K, and Ca, respectively. Although the RMSE for NO_3_ was higher compared to other ISEs, this can be attributed to the higher tested range of NO_3_ concentrations than other ions. Moreover, considering that NO_3_ has a higher equivalent weight than other ions (i.e., NO_3_: 62 mg/me, K: 39 mg/me, Ca: 40mg/me), the ISEs applied in the system demonstrated sufficient applicability for the ions in the hydroponic nutrient solution.

### 3.2 Five-step replenishment test

**Figure 4:**
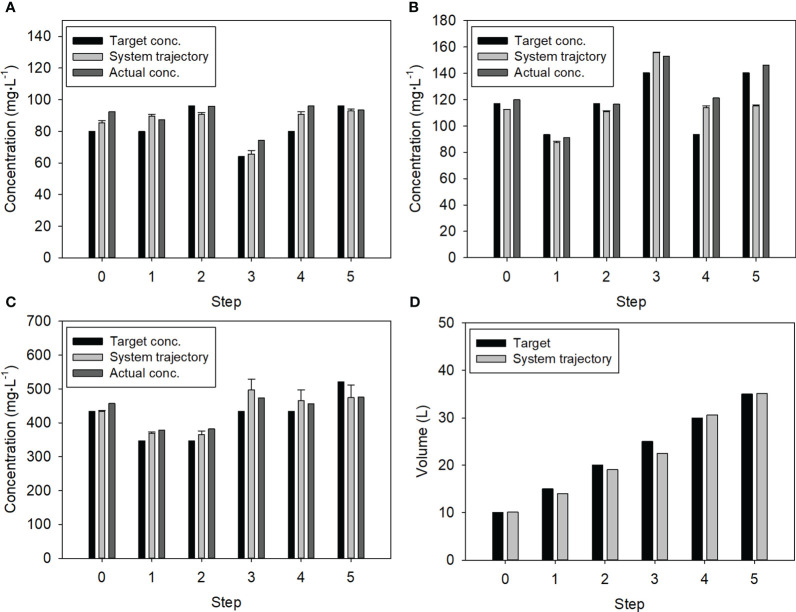
Changes in ion concentrations and nutrient solution volume for the stepwise test: **(A)** Ca; **(B)** K; **(C)** NO_3_; **(D)** Nutrient solution volume. Error bars denote the standard deviation of the multiple ISEs for NO_3_, K, and Ca.

Considering the target concentrations for the five steps, the system realized replenishment based on the developed dosing algorithm, and the NO_3_, K, and Ca ions in the resulting solutions were measured by the system and standard analyzers (**Figure 4**). These replenishments began with an initial solution having higher concentrations than the target values. The system trajectory indicated the ion concentrations measured by the ISEs of the system.

In the case of the Ca concentration, over-injection occurred in the 3^rd^ step, resulting in a 13.6% higher concentration in the 4^th^ step (**Figure 4A**). When the Ca concentration was accurately measured in the 4^th^ step, it closely followed the target concentration in the next step. Moreover, the high K concentration in the 3^rd^ step helped maintain the high concentration level in the 4^th^ step (**Figure 4B**). The K concentration and volume measured by the system in the 3^rd^ step were 155.7 mg·L^-1^ and 22.52 L, respectively. This led to an 11% underestimation of the K concentration in the 5^th^ step. Except for these cases, the NO_3_ concentrations and nutrient solution volumes closely followed the target values (**Figures 4C, D**).

Overall, the Ca, K, and NO_3_ concentrations and volume of the nutrient solution were controlled with average relative errors of 10.6 ± 8.0%, 7.9 ± 2.1%, 8.0 ± 11.0%, and 4.2 ± 3.7%, respectively, during the stepwise test.

### 3.3 Comparative analysis of the dosing algorithms

The decision-tree method and simplex method were used to calculate the amounts of fertilizer salts to be added, based on the time log of the fertilizer pumps and measured ion concentrations. These values were then compared (**Table 2**).

**Table 2:** Amounts of the fertilizer salts to add determined by the simplex method and the decision-tree method for the five-stepwise test.

Step	Simplex method								Decision-tree method							
	Injected salts (mg)							Minor (ml)	Injected salts (mg)							Minor (ml)
	Ca(NO_3_)_2_·4H_2_O	KH_2_PO_4_	NH_4_H_2_PO_4_	KNO_3_	NH_4_NO_3_	MgSO_4_·7H_2_O	K_2_SO_4_		Ca(NO_3_)_2_·4H_2_O	KH_2_PO_4_	NH_4_H_2_PO_4_	KNO_3_	NH_4_NO_3_	MgSO_4_·7H_2_O	K_2_SO_4_	
1^st^	1983	0	223.8	0	0	1618.4	853.2	24.4	1983	81.8	152.5	0	0	1618.4	541.5	24.4
2^nd^	3946.7	0	337.5	0	0	557.1	2928.4	30.15	3946.7	99.9	207.9	0	0	557.1	2428.8	30.15
3^rd^	0	1626.5	0	4565.6	1611.6	842.8	0	29.7	0	0	21.3	4778.8	1142.8	842.8	0	29.7
4^th^	5443.2	0	699.1	0	0	1971.8	1353.1	37.4	5443.2	89.2	339.7	0	0	1971.8	0	37.4
5^th^	3412.9	901.2	0	2482.5	870.7	1066.7	474.2	21.9	3412.9	0	252	3582.3	0	1066.7	103.1	21.9
Total^*^	14785.8	2527.7	1260.4	7048.1	2482.3	6056.4	5608.9	143.55	14785.8	270.9	973.4	8361.1	1142.8	6056.4	3073.4	143.55
Total^**^	39769.6							143.55	34663.8							143.55

^*^Total amounts for each fertilizer salts.

^**^Total amounts for all fertilizer salts.

The required volumes of the concentrated solution for minor elements were identical because they were determined according to the water volume to be added. Similarly, the determined amounts of Ca(NO_3_)_2_·4H_2_O and MgSO_4_·7H_2_O were also identical for the simplex method and decision tree method. The algorithms yielded different values for the required amounts of KH_2_PO_4_, NH_4_H_2_PO_4_, KNO_3_, NH_4_NO_3_, and K_2_SO_4_. Specifically, although the simplex method recommended the use of less KNO_3_ than the decision tree method, it recommended the use of more KH_2_PO_4_, NH_4_H_2_PO_4_, NH_4_NO_3_, and K_2_SO_4_ salts compared with the decision tree method. Consequently, the total amounts of the fertilizer salts to be added were 14.7% higher in the simplex method than the decision tree method. Zeros in the injected salts indicate situations where the salts were not necessary, or the calculated injection mass was less than zero.

**Figure 5:**
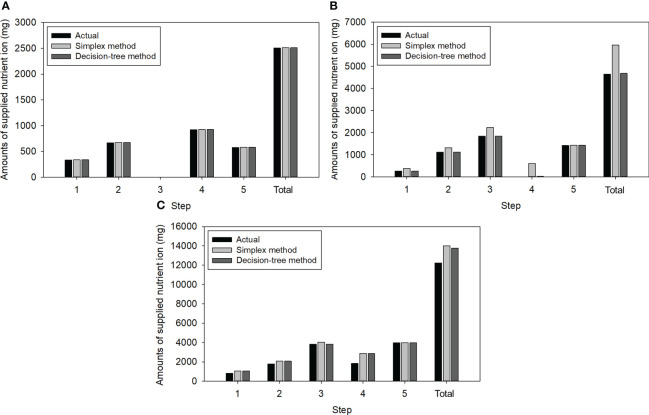
Amounts of the three nutrient ions required for the five-stepwise replenishment: **(A)** Ca; **(B)** K; **(C)** NO_3_.

**Figure 5** shows the resulting amounts of NO_3_, K, and Ca ions to be added, as determined by the simplex method and decision tree method, in comparison with the actual required ion mass. The calculated amounts for Ca ions were identical to the required amounts, indicating that both methods could achieve a complete solution (**Figure 5A**). A notable difference was observed in the case of K ions (**Figure 5B**). Both methods reduced the overdose of K ions, as the K ions present in the 3^rd^ step solution were comparable to the expected K ions in the 4^th^ step solution. However, the decision tree method could more accurately control the K ions than the simplex method at other steps, thereby reducing 22% of the total K injection for the test. Over-injections of NO_3_ ions were observed in both methods due to the coupling of NO_3_ with Ca (**Figure 5C**). Specifically, the amounts were 1.7% higher in the simplex method due to NH_4_NO_3_ injections (**Table 2**; **Figure 5C**).

### 3.4 Evaluation of carbon dioxide emissions

**Figure 6:**
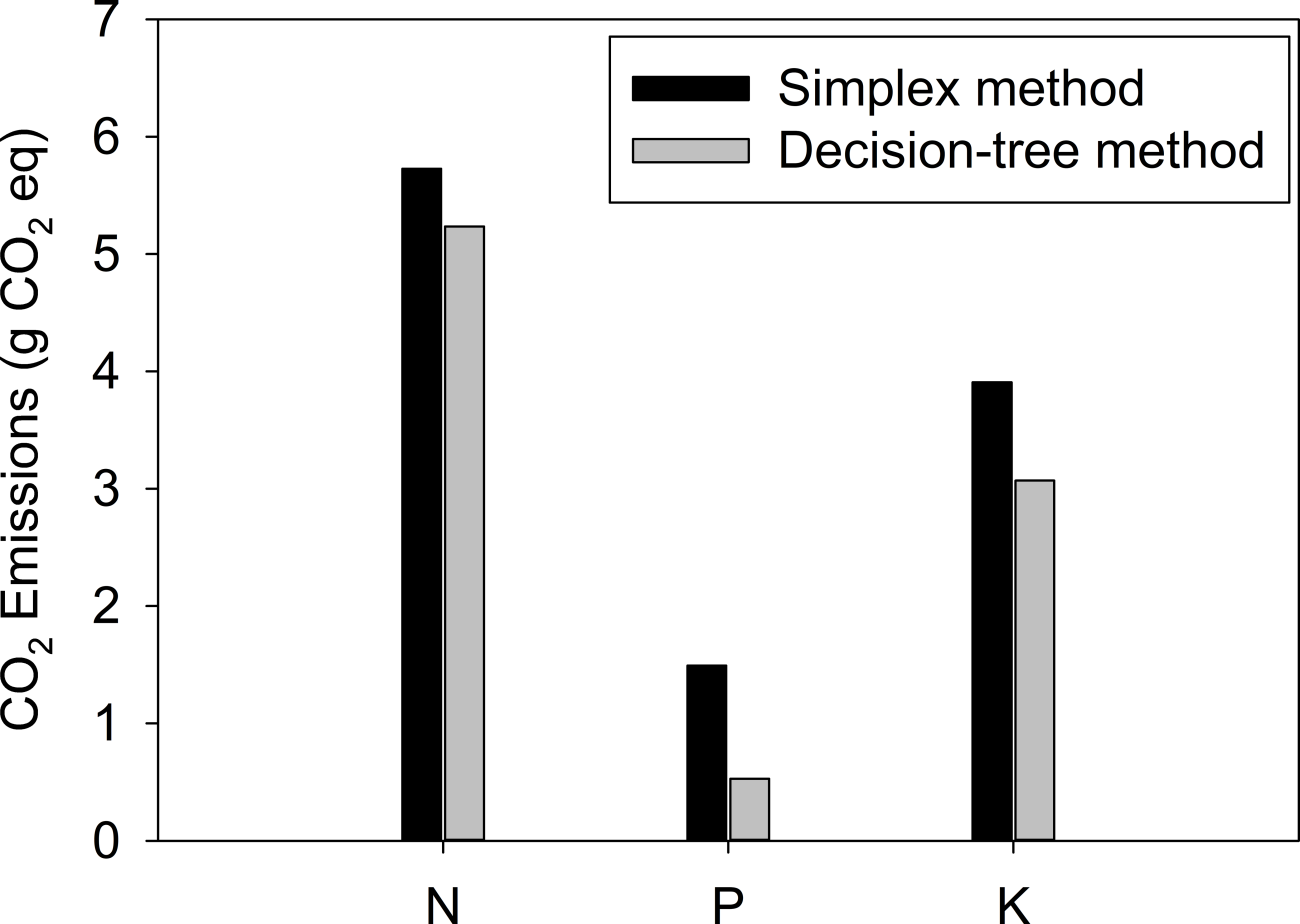
Carbon dioxide emissions from different types of fertilizer dosing algorithms.

**Figure 6** presents the calculated carbon dioxide equivalents for the two dosing algorithms. Differences were observed in the carbon dioxide emissions from two ion-specific dosing algorithms. Specifically, the carbon dioxide equivalents for N, P, and K from the simplex method were 9.4%, 182.5%, 27.3% higher than those from the decision tree method, respectively.

## 4 Discussion

Various researchers have focused on ion-specific management in closed hydroponic solutions. Improving the accuracies of ion-specific nutrient management can promote more efficient and sustainable agricultural practices. Accordingly, in recent studies, there have been reports on attempts to apply spectroscopy-based monitoring technologies for accurate and rapid measurement of ion concentrations in nutrient solutions (Stevens et al., 2023; Sulaiman et al., 2024). The developed technologies can measure ions that are currently challenging to measure with ISE. However, there is still much room for improvement in terms of cost and lifespan. Moreover, there is a scarcity of information on how to adjust the dosage of fertilizers using the measured ion concentrations, despite efforts to predict concentrations through artificial intelligence (Tuan et al., 2021). Until the latest researches, researchers have, for the most part, conducted fertilizer dosing calculations through simplex method, considering only limited ion scenarios (Gieling et al., 2005; Jung et al., 2015; Xu et al., 2020; Chowdhury et al., 2021). Although the simplex method is a simple and powerful method for finding solutions with various combinations of nutrient salts, it may yield negative solutions. Even when the solutions are restricted to positive ranges, inaccurate nutrient supplementation may occur. These issues may aggravate when the reliabilities of the ion sensors and pumps are inadequate, or a limited number of fertilizer salts are used.

In this study, the performance test demonstrated that the ISEs equipped in the system can provide accurate information regarding the individual ion concentrations in the hydroponic nutrient solutions (**Figure 3**). The performance of the ISEs in the hydroponic solutions is consistent with those reported previously (Kim et al., 2013; Vardar et al., 2015; Cho et al., 2017; Jung et al., 2019; Kim et al., 2023). However, the accuracy is not ideal and has remained largely unchanged for decades. Therefore, more advanced dosing algorithms for ion-specific nutrient management must be established.

Notably, the decision tree algorithm proposed in this study does not compute the exact solution but rather determines appropriate solution considering the correlations among single-nutrient fertilizers. It effectively minimizes the under- or over-injection of nutrient ions based on the preset priority of nutrient ions. This effectiveness was confirmed through the five-stepwise test (**Figure 4**, **Table 2**). The decision tree algorithm variably controlled the individual fertilizers for attaining the target concentration levels. By assessing various combinations of fertilizer injection, the algorithm computed the appropriate mass to be added for each fertilizer salt, thereby preventing unnecessary salt injections (**Table 2**). For example, in the 3^rd^ step, Ca(NO_3_)_2_·4H_2_O fertilizer was not used because there were sufficient Ca ions for the next target level. Similarly, the algorithm used Ca(NO_3_)_2_·4H_2_O, K_2_SO_4_, and NH_4_H_2_PO_4_, to supply NO_3_, K, NH_4_, and H_2_PO_4_ ions for the 1^st^, 2^nd^, and 4^th^ steps, instead of KH_2_PO_4_, KNO_3_ and NH_4_NO_3_. However, in the 3^rd^ stage, a completely different combination was used. These results support that the proposed dosing algorithm is more efficient compared with the conventional algorithm that cannot prevent unnecessary ion injections (Ko et al., 2013; Moon et al., 2019).

Although several instances of under- or over-injection were observed, it is considered acceptable for management performance to remain within a 10% margin, given that practical hydroponic ion concentrations may fluctuate by more than 50% (Ahn et al., 2021). Furthermore, the ion-specific nutrient management system can be improved by adopting more accurate ion sensors, pumps, and agitators for mixing the nutrient solution. Additionally, the closed-loop control logic can be further improved (Li and Dixon, 2016).

The decision tree method and simplex method yielded different fertilizer dosages (**Table 2**). The dosing amounts calculated using the simplex method sometimes included negative values to formulate a complete solution for the given target concentrations (Kitahara and Mizuno, 2013). However, negative dosing for specific ions is not feasible because nutrient ions cannot be selectively removed in general hydroponic systems. Therefore, the pump operation times were merely indicated as zero. However, the other salts, including those with the same ions as the nutrient salts, had negative dosing amounts, necessitating larger replenishments to compensate for the ion deficits. Although the simplex method could be modified to calculate an approximate solution consisting of only positive values, it would induce more unbalanced injections. In contrast, the decision-tree method could effectively minimize over-injection by adjusting the injection mass based on the preset nutrient priority. Specifically, the decision tree method enabled more accurate control of K ions than the simplex method at other steps because of the negative values yielded by the simplex method, as discussed (**Figure 5B**). Moreover, when the simplex method was used, KH_2_PO_4_ and NH_4_H_2_PO_4_ were over-injected to offset the negative values of ions from the complete solution (**Table 2**). Consequently, N, P, and K were administered in larger quantities using the simplex algorithm compared with the decision-tree-based algorithm. Such inefficient fertilizer injections ultimately lead to the generation of more greenhouse gases.

Although it is in the early stages, to the best of my knowledge, this paper is among the first to propose the possibility of reducing greenhouse gas emissions by improving the fertilizer dosing algorithm. The equivalent emissions computed in this study may vary across countries or companies (Brentrup et al., 2018), but they can be easily adjusted by incorporating the CO_2_ equivalents into the conversion formula. The findings of this study demonstrate that the proposed dosing algorithm can not only enhance the nutrient composition of recycled hydroponic solutions but also contribute to sustainability (**Figure 6**). However, this study has several significant limitations. Firstly, the algorithm validation was conducted not in an actual crop cultivation environment but rather focused on accurately tracking specific ion concentration settings. Secondly, the stability of nutrient management was not verified for ions not measured by the sensors. Lastly, the study did not address whether ion-based control leads to actual fertilizer savings and a subsequent reduction in carbon emissions compared to conventional EC-based closed hydroponic solution management. In future research, our objective is to conduct crop cultivation experiments using the decision-tree-based nutrient solution control system. The focus of future research efforts will be on comparing the performance and stability of the algorithm with the conventional recirculating cultivation method based on EC. Additionally, we plan to verify fertilizer usage efficiency and assess the consequent reduction in carbon emissions.

## 5 Conclusions

A decision-tree-based dosing algorithm for closed hydroponic solution was developed and applied to an automated ion-specific nutrient management system with an array of NO_3_, K, and Ca ISEs. The performance of the proposed algorithm was evaluated through a five-step replenishment test and compared with that of the conventional simplex algorithm in terms of the replenishment accuracy and carbon dioxide emissions. From the results, the proposed algorithm exhibited high fertilizer efficiency and reduced carbon emission.

The results highlighted the proposed algorithm effectively controlled individual ion concentrations in the nutrient solution by using the ion concentrations measured by an array of ISEs and the decision-tree-based nutrient dosing algorithm. Specifically, the proposed system minimized the overdose of fertilizers if specific ion levels were higher than the target values. Although the simplex algorithm performed well when no negative values were present in the complete solutions, the fertilizers were sometimes over-injected when negative values were present. Consequently, the decision tree method led to more effective dosing than the simplex method by reducing approximately 13% of the total fertilizer inputs during the stepwise replenishment test. Furthermore, efficient fertilizer application led to an 8.6% reduction in carbon dioxide emissions for N, a 64.6% reduction for P, and a 21.4% reduction for K.

These findings suggest that the decision tree algorithm is an efficient alternative for managing closed hydroponic solutions while reducing carbon dioxide emissions, thereby promoting sustainable crop production.

## Data availability statement

The original contributions presented in the study are included in the article/**Supplementary Material**. Further inquiries can be directed to the corresponding author.

## Author contributions

W-JC: Conceptualization, Formal Analysis, Investigation, Methodology, Writing – original draft, Writing – review & editing. M-SG: Investigation, Methodology, Writing – original draft, Writing – review & editing. D-WK: Data curation, Formal Analysis, Writing – review & editing. JK: Data curation, Formal Analysis, Writing – review & editing. D-HJ: Conceptualization, Formal Analysis, Methodology, Writing – review & editing. H-JK: Funding acquisition, Supervision, Writing – review & editing.

## References

[B1] Ahn T. I. Shin J. H. Son J. E. (2021). Theoretical and experimental analyses of nutrient control in electrical conductivity-based nutrient recycling soilless culture system. Front. Plant Sci. 12, 656403. doi: 10.3389/fpls.2021.656403 34108979 PMC8181128

[B2] Bamsey M. Graham T. Thompson C. Berinstain A. Scott A. Dixon M. (2012). Ion-specific nutrient management in closed systems: the necessity for ion-selective sensors in terrestrial and space-based agriculture and water management systems. Sensors 12, 13349–13392. doi: 10.3390/s121013349 23201999 PMC3545570

[B3] Brentrup F. Lammel J. Stephani T. Christensen B. (2018). Updated carbon footprint values for mineral fertilizer from different world regions. Proc.11th Int. Conf. on Life Cycle Assess. of Food. (Bangkok, Thailand: KU-JGSEE-NSTDA-FTI) 17–19.

[B4] Cho W.-J. Kim H.-J. Jung D.-H. Han H.-J. Cho Y.-Y. (2019). Hybrid signal-processing method based on neural network for prediction of NO3, K, ca, and mg ions in hydroponic solutions using an array of ion-selective electrodes. Sensors 19, 5508. doi: 10.3390/s19245508 31847136 PMC6960818

[B5] Cho W. J. Kim H. J. Jung D. H. Kang C. I. Choi G. L. Son J. E. (2017). An embedded system for automated hydroponic nutrient solution management. Trans. Asabe 60, 1083–1096. doi: 10.13031/trans.12163

[B6] Chowdhury M. Islam M. N. Reza M. N. Ali M. Rasool K. Kiraga S.. (2021). Sensor-based nutrient recirculation for aeroponic lettuce cultivation. J. Biosyst. Eng. 46, 81–92. doi: 10.1007/s42853-021-00089-8

[B7] De Rijck G. Schrevens E. (1994). Application of mixture-theory for the optimisation of the composition of the nutrient solution". Acta Hortic. 401, 283–292. doi: 10.17660/ActaHortic.1995.401.34

[B8] Domingues D. S. Takahashi H. W. Camara C. Nixdorf S. L. (2012). Automated system developed to control pH and concentration of nutrient solution evaluated in hydroponic lettuce production. Comput. Electron. Agric. 84, 53–61. doi: 10.1016/j.compag.2012.02.006

[B9] Geoffrey R. Dixon M. A. Arnold K. E. (1997). Evaluation of sensor technologies for automated control of nutrient solutions in life support systems using higher plants. Proc. 6th European Symp. on Space Environ. Control Syst. (Noordwijk, Netherlands: European Space Agency), 851–858.

[B10] Gieling T. H. Van Straten G. Janssen H. J. J. Wouters H. (2005). ISE and chemfet sensors in greenhouse cultivation. Sensors Actuators B-Chemical 105, 74–80. doi: 10.1016/S0925-4005(04)00113-3

[B11] Hoagland D. R. Arnon D. I. (1950). The water-culture method for growing plants without soil. Circular. Circular. California Agric. Experiment Station 347 (2nd edit). p.32.

[B12] Jung D.-H. Kim H.-J. Cho W.-J. Park S. H. Yang S.-H. (2019). Validation testing of an ion-specific sensing and control system for precision hydroponic macronutrient management. Comput. Electron. Agric. 156, 660–668. doi: 10.1016/j.compag.2018.12.025

[B13] Jung D. H. Kim H. J. Choi G. L. Ahn T. I. Son J. E. Sudduth K. A. (2015). Automated lettuce nutrient solution management using an array of ion-selective electrodes. Trans. Asabe 58, 1309–1319. doi: 10.13031/trans.58.11228

[B14] Katsoulas N. Savvas D. Kitta E. Bartzanas T. Kittas C. (2015). Extension and evaluation of a model for automatic drainage solution management in tomato crops grown in semi-closed hydroponic systems. Comput. Electron. Agric. 113, 61–71. doi: 10.1016/j.compag.2015.01.014

[B15] Kim H. J. Kim W. K. Roh M. Y. Kang C. I. Park J. M. Sudduth K. A. (2013). Automated sensing of hydroponic macronutrients using a computer-controlled system with an array of ion-selective electrodes. Comput. Electron. Agric. 93, 46–54. doi: 10.1016/j.compag.2013.01.011

[B16] Kim J. Kim H.-J. Gang M.-S. Kim D.-W. Cho W.-J. Jang J. K. (2023). Closed hydroponic nutrient solution management using multiple water sources. J. Biosyst. Eng. 48, 215–214. doi: 10.1007/s42853-023-00182-0

[B17] Kitahara T. Mizuno S. (2013). A bound for the number of different basic solutions generated by the simplex method. Math. Programming 137, 579–586. doi: 10.1007/s10107-011-0482-y

[B18] Klespitz J. Kovács L. (2014). Peristaltic pumps—A review on working and control possibilities. Proc. IEEE 12th Int. Symp. on Appl. Machine Intelligence and Inform. (Herl'any, Slovakia: IEEE), 191–194. doi: 10.1109/SAMI.2014.6822404

[B19] Ko M. T. Ahn T. I. Son J. E. (2013). Comparisons of ion balance, fruit yield, water, and fertilizer use efficiencies in open and closed soilless culture of paprika (Capsicum annuum L.). Korean J. Hortic. Sci. Technol. 31, 423–428. doi: 10.7235/hort.2013.13028

[B20] Kozai T. Tsukagoshi S. Sakaguchi S. (2018). Toward nutrient solution composition control in hydroponic system. In: Kozai T. (eds) Smart Plant Factory (Singapore: Springer), 395–403. doi: 10.1007/978-981-13-1065-2_24

[B21] Li Z. Dixon S. (2016). A closed-loop operation to improve GMR sensor accuracy. IEEE Sensors J. 16, 6003–6007. doi: 10.1109/JSEN.2016.2580742

[B22] Moon T. Ahn T. I. Son J. E. (2019). Long short-term memory for a model-free estimation of macronutrient ion concentrations of root-zone in closed-loop soilless cultures. Plant Methods 15, 1–12. doi: 10.1186/s13007-019-0443-7 31160918 PMC6540585

[B23] Namazkhan M. Albers C. Steg L. (2020). A decision tree method for explaining household gas consumption: The role of building characteristics, socio-demographic variables, psychological factors and household behaviour. Renewable Sustain. Energy Rev. 119, 109542. doi: 10.1016/j.rser.2019.109542

[B24] Neocleous D. Savvas D. (2016). NaCl accumulation and macronutrient uptake by a melon crop in a closed hydroponic system in relation to water uptake. Agric. Water Manage. 165, 22–32. doi: 10.1016/j.agwat.2015.11.013

[B25] Neocleous D. Savvas D. (2022). Validating a smart nutrient solution replenishment strategy to save water and nutrients in hydroponic crops. Front. Environ. Sci. 10, 965964. doi: 10.3389/fenvs.2022.965964

[B26] Resh H. M. (2016). Hydroponic food production: a definitive guidebook for the advanced home gardener and the commercial hydroponic grower (7th ed). (Boca Raton, FL: CRC Press).

[B27] Rius-Ruiz F. X. Andrade F. J. Riu J. Rius F. X. (2014). Computer-operated analytical platform for the determination of nutrients in hydroponic systems. Food Chem. 147, 92–97. doi: 10.1016/j.foodchem.2013.09.114 24206690

[B28] Sambo P. Nicoletto C. Giro A. Pii Y. Valentinuzzi F. Mimmo T.. (2019). Hydroponic solutions for soilless production systems: Issues and opportunities in a smart agriculture perspective. Front. Plant Sci. 10. doi: 10.3389/fpls.2019.00923 PMC6668597 31396245

[B29] Shiyab S. M. Shatnawi M. A. Shibli R. A. Al Smeirat N. G. Ayad J. Akash M. W. (2013). Growth, nutrient acquisition, and physiological responses of hydroponic grown tomato to sodium chloride salt induced stress. J. Plant Nutr. 36, 665–676. doi: 10.1080/01904167.2012.754037

[B30] Son J. E. Kim H. J. Ahn T. I. (2020). “Chapter 20 - hydroponic systems,” in Plant factory, 2nd ed. Eds. Kozai T. Niu G. Takagaki M. (London: Academic Press), 273–283. doi: 10.1016/C2018-0-00969-X

[B31] Sonneveld C. Voogt W. Spaans L. (1999). A universal algorithm for calculation of nutrient solutions. Acta Hortic. 481, 331–340. doi: 10.17660/ActaHortic.1999.481.38

[B32] Stevens J. D. Murray D. Diepeveen D. Toohey D. (2023). Development and testing of an ioT spectroscopic nutrient monitoring system for use in micro indoor smart hydroponics. Horticulturae 9, 185. doi: 10.3390/horticulturae9020185

[B33] Sulaiman R. Azeman N. H. Mokhtar M. H. H. Mobarak N. N. Abu Bakar M. H. Bakar A. (2024). Hybrid ensemble-based machine learning model for predicting phosphorus concentrations in hydroponic solution. Spectrochimica Acta Part A: Mol. Biomolecular Spectrosc. 304, 123327. doi: 10.1016/j.saa.2023.123327 37708761

[B34] Tuan V. N. Dinh T. D. Zhang W. Khattak A. M. Le A. T. Saeed I. A.. (2021). A smart diagnostic tool based on deep kernel learning for on-site determination of phosphate, calcium, and magnesium concentration in a hydroponic system. RSC Adv. 11, 11177–11191. doi: 10.1039/D1RA00140J 35423630 PMC8695829

[B35] Vardar G. Altıkatoğlu M. Ortaç D. Cemek M. (2015). Measuring calcium, potassium, and nitrate in plant nutrient solutions using ion-selective electrodes in hydroponic greenhouse of some vegetables. Biotechnol. Appl. Biochem. 62, 663–668. doi: 10.1002/bab.1317 25388287

[B36] Wang Z.-B. Chen J. Mao S.-C. Han Y.-C. Chen F. Zhang L.-F.. (2017). Comparison of greenhouse gas emissions of chemical fertilizer types in China's crop production. J. Cleaner Production 141, 1267–1274. doi: 10.1016/j.jclepro.2016.09.120

[B37] Xu K. Kitazumi Y. Kano K. Shirai O. (2019). Construction of an automatic nutrient solution management system for hydroponics-adjustment of the K+-Concentration and volume of water. Analytical Sci. 35, 595–598. doi: 10.2116/analsci.18A003 30662013

[B38] Xu K. Kitazumi Y. Kano K. Shirai O. (2020). Automatic management of nutrient solution for hydroponics-construction of multi-ion stat. Anal. Sci. 36, 1141–1144. doi: 10.2116/analsci.20A002 32307344

