# Supplementary material for: Decision-tree-based ion-specific dosing algorithm for enhancing closed hydroponic efficiency and reducing carbon emissions
Source: Front Plant Sci. 2023 Dec 18;14:1301490. doi: 10.3389/fpls.2023.1301490 (PMC10757981; doi:10.3389/fpls.2023.1301490)
Supplement: Supplementary file 1 [file Table_1.docx]

Supplementary Material

**Supplementary Table 1.** Ion concentrations of the solutions used in the experiment (Unit: mg∙L^−1^).

| Solution | Concerned ion | Concentration (mg∙L^-1^) |
| --- | --- | --- |
| Two-point normalization solution (high) | Ca | 248.03 |
|  | K | 308.91 |
|  | NO_3_ | 981.7 |
| Two-point normalization solution (low) | Ca | 28.37 |
|  | K | 35.39 |
|  | NO_3_ | 100.9 |
| Stock solution 1 (Ca(NO_3_)_2_∙4H_2_O) | Ca | 3489.11 |
|  | NO_3_ | 11243.6 |
| Stock solution 2 (KH_2_PO_4_) | K | 6450.5 |
|  | P | 4149.582 |
| Stock solution 3 (NH_4_H_2_PO_4_) | NH_4_ | 3340.28 |
|  | P | 4732.319 |
| Stock solution 4 (KNO_3_) | K | 7627.55 |
|  | NO_3_ | 13564.3 |
| Stock solution 5 (NH_4_NO_3_) | NH_4_ | 4430.25 |
|  | NO_3_ | 16608 |
| Stock solution 6 (MgSO_4_∙7H_2_O) | Mg | 2071.4 |
| Stock solution 7 (K_2_SO_4_) | K | 8857.83 |
| pH control solution (H_2_SO_4_) | H | pH 2.0 |
| Tap water | Ca | 16.13-23.4 |
|  | K | 2.66-3.51 |
|  | NO_3_ | 5.9-9.2 |
|  | P | 0.113-0.136 |
|  | Mg | 3.24-4.77 |
|  | NH_4_ | Not detected |
|  | SO_4_ | 3.24-15.6 |

Supplementary Table 2.. Specifications of components of the ion-specific management system.

| Component | Specification | Manufacturer/Model |
| --- | --- | --- |
| Sample chamber | A chamber of Poly(methyl methacrylate): 100 mL | Megascience (Seoul, South Korea), Sensor chamber |
| Sensor array | K ISE  Measuring range: 3-700 mg·L^-1^  Detection limit: 3  Response time: ~50s  NO_3_ ISE  Measuring range: 3-1600 mg·L^-1^  Detection limit: 3  Response time: ~50s  Ca ISE  Measuring range: 3-700 mg·L^-1^  Detection limit: 3  Response time: ~50s  Reference electrode: Double-junction | K, NO_3_: Laboratory-made  Ca: Thermo Fisher Scientific (MA, USA), 9320BN  Reference: Thermo Fisher Scientific (MA, USA), 900200 |
| Sampling & drainage pumps | Peristaltic pump  Flow rate: 0.22 L·min^-1^  Tubing material: Silicon  Inner tubing diameter: 1.6 mm  Power: 24 VDC | ASF THOMAS (Puchheim, Germany), SR10/50 |
| Stock solution pumps | Peristaltic pump  Flow rate: 0.1 L·min^-1^  Tubing material: Novoprene  Inner tubing diameter: 1.6 mm  Power: 24 VDC | ASF THOMAS (Puchheim, Germany), SR10/50 |
| Water replenishment pump | Peristaltic pump  Flow rate: 0.525 L·min^-1^  Tubing material: Silicon  Inner tubing diameter: 4.8 mm  Power: 24 VDC | BOXER (Ottobeuren, Germany), QQ15 |
| Nutrient solution supplying pump | Centrifugal pump  Flow rate: 33.3 L∙min^-1^  Maximum pressure height: 10.19 kgf∙cm^-2^  Power: 3PH 380 VAC | Hwarang System Co., Ltd. (Incheon, South Korea), PP50Y |
| Main control system | CPU: 3.4 GHz (i7 4770, Intel)  Memory: DDR3 8gb  OS: Window 7  Main program: LabVIEW (v2015, National Instruments, TX, USA) | Hewlett-Packard (CA, USA), EliteDesk 800 G1 TWR |
| Solution tanks | Two-point normalization solutions (5 L for each) | Korea First Safety (Incheon, South Korea), 5L HDPE (high density polyethylene) tank |
|  | Stock solutions (2 L for each) | Korea First Safety (Incheon, South Korea), 2L HDPE (high density polyethylene) water tank |
|  | Nutrient solution mixing tank (Max. 100 L) | Bestplastic (Gyeonggi-do, South Korea), 100L PE (polyethylene) water tank |
| Water-level sensor | Reflective Ultrasonic Level Transmitters  Measurement range: 0.038-1.5 m  Automatic temperature compensation: -40-80 °C  Signal output range: 4-20 mA  Power: 24 VDC | Flowline, Inc. (CA, USA), EchoPod UG01 |
| Data acquisition board | A/D converter for ISE signals  Input channel: 16 bit analog input  Sampling rate: 250 kS∙s^-1^ | National Instrument (TX, USA), PCI-6221 |
| Signal conditioner | Isolated analog input board for ISEs  Input range: ± 10 V  Gain: 1 | National Instrument (TX, USA), SCC-AI13 |
| Relay | Solid state relay  Input voltage range: 0~60 VDC  Output voltage range: 0~60 VDC  Channel: 8 ch. | National Instrument (TX, USA), NI-9485 |
